# Production of Al Alloys with Kelvin Cells Using the Lost-PLA Technique and Their Mechanical Characterization via Compression Tests

**DOI:** 10.3390/ma18020296

**Published:** 2025-01-10

**Authors:** Alessandra Ceci, Corrado Cerini, Girolamo Costanza, Maria Elisa Tata

**Affiliations:** Industrial Engineering Department, University of Rome Tor Vergata, 00133 Rome, Italy; alessandra.ceci@uniroma2.it (A.C.); corrado_cerini@libero.it (C.C.); elisa.tata@uniroma2.it (M.E.T.)

**Keywords:** 3D cellular structures, Al foams, Kelvin cell, mechanical properties, compression behavior, experimental tests

## Abstract

The mechanical behavior of AA6082 Kelvin cell foams under compressive tests has been investigated in this work. The lost-PLA replication technique, a simple and cheap technique, has been adopted as the production method. Six Al alloy samples have been made and successively subjected to compressive tests in order to examine the mechanical response and the repeatability too. The manufactured foams show good morphology and surface finishing, replicating the PLA 3D-printed foams with adequate accuracy. The experimental density of the foam has been found in good agreement with the theoretical one. When subjected to static compression, the Kelvin cell foams exhibit a load–strain diagram characterized by the initial linear stage followed by two plateaus at successively increasing load levels. Final densification occurs when there is no more space available for further plastic deformation and the load sharply increases. The specific absorbed energy has been calculated from load–strain curves: the average measured value was found to be 2.3 J/cm^3^, and standard deviation in the six compression tests was 0.3 J/cm^3^.

## 1. Introduction

The need for lightweight structures that guarantee high mechanical strength and superior overall performance is continuously rising in various engineering fields [1,2], both in static and dynamic conditions [3]. Accurately controlling the porosity’s materials [4,5,6] is fundamental in achieving and combining these apparently contrasting requirements [7,8,9]. Thanks to additive manufacturing technologies [10,11], it is possible to manage and built integrated components entirely or partially made of lattice cellular structures with suitable and optimized geometries [12,13,14]. The transportation [15,16] and civil engineering [17,18] fields have benefited mainly from the chance to create free-form and lightweight components, in which weight reduction is fundamental. The high energy absorption capacity [19,20] and the previously mentioned advantage of weight reduction [21,22] make these structures interesting for the automotive sector, where they are also used as acoustic dampers [23,24]. The high surface area that the porosity generates also guarantees excellent properties in the field of heat exchange to the metal foams. Managing a lattice cellular structure with optimized geometry is a complex task [25,26,27]. However, additive technologies represent the state of the art in the manufacturing of cellular metallic structures thanks to their incredible versatility of use [28,29] and their outstanding mechanical properties [30,31,32].

Metal cellular structures are materials exhibiting a controlled level of porosity; they can be made of a network of interconnected cells or voids. A first kind of classification [33] may be based on the geometry and morphology of the microstructures. Cellular materials can be divided in two main categories: stochastic structures, usually known as metallic foams (structures with open or closed cells) [9] and non-stochastic structures [34], obtained by periodic repetition in the space of lattice structures [35,36]. In the initial scenario, the porosity of the distribution is random, whereas in the latter case, it shows periodic characteristics. Numerous studies indicate that utilizing non-stochastic open-cell structures can result in enhanced and predictable mechanical and thermal characteristics in comparison to stochastic foams [37,38]. Hassanli et al. [20] demonstrated that a correct modification in pore distribution can significantly improve the mechanical properties of the fabricated foam. The elevated surface-to-volume ratio renders this type of material ideal for the production of high-performance heat exchangers [39,40,41]. Research has demonstrated that periodic cellular structures provide a heat transfer coefficient that outperforms that of metal foams [42,43,44].

A simple and economical approach for producing metal porous structures, suitable for both functional and structural purposes, with the predetermined design of a periodic cell, has been demonstrated in earlier research [14]. The main processing steps are reported in the following. Beginning with a 3D computer model (CAD), the part is fabricated using fused deposition modeling (FDM), which allows for the utilization of thermoplastic materials (PC, ABS, and PLA) that have relatively low melting points. In the FDM printer, the filament is heated until it reaches a semi-liquid state near the nozzle and is then extruded layer by layer onto the moving platform. Each successive layer bonds with the previous one until the final component solidifies. Several parameters of the process, including layer thickness, printing orientation, raster width, and angle, can be adjusted to enhance the overall quality of the printed pieces. The set-up is characterized by its low cost, high speed, and ease of use.

The manufacturing of the metallic lattice structure is performed through the replication of the PLA model in a similar way as used in the past, such as in lost wax casting. However, Poly Lactic Acid (PLA), a biodegradable polymer, is employed instead of wax. Once the printing process is completed, the PLA model, placed inside a mold, is filled up with liquid plaster. After plaster drying, the PLA is removed in a suitable furnace with a burnout process. In the next step, molten Al alloys replace the voids left free by the PLA, followed by alloy solidification. After that, the plaster can be easily removed, and finally, the lattice structure replicating the starting geometry of the printed PLA can be obtained. Thanks to the experience gained using the technique, many problems have been solved, as detailed in an earlier work [14]. The materials and experimental techniques used are described in detail in the next paragraph.

This research work analyzes the manufacturing process of metallic lattice structures with a Kelvin cell, and after production, their mechanical characterization was also analyzed through compression tests. It deals with the production of EN AW 6082 cellular structures manufactured by the lost-PLA technique. The aim of the work is the analysis of the energy absorption capability of Al metal foams with a predetermined structure (Kelvin cells).

## 2. Materials and Methods

**Figure 1:**
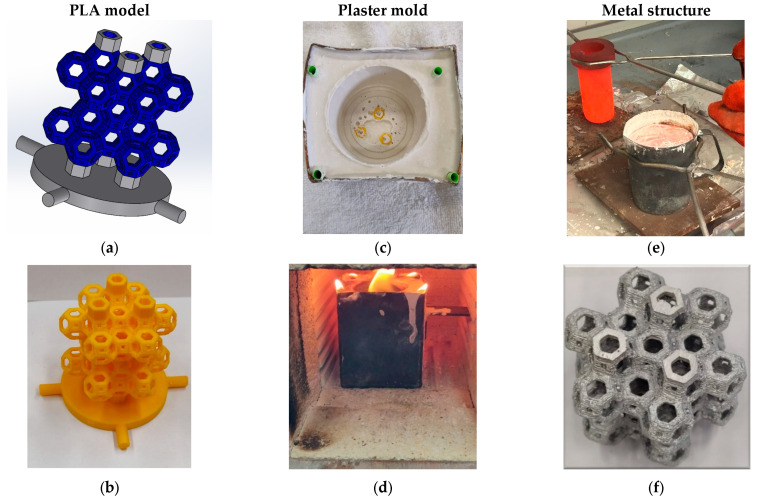
(**a**) CAD model; (**b**) 3D-printed PLA model; (**c**) plaster casting; (**d**) burnout; (**e**) gravity casting; (**f**) final AA 6082 structure.

The lost-PLA technique was derived from a foundry process well known in ancient times as lost wax casting. In particular, to adopt the lost-PLA technique, a 3D model of the structure needs to be produced. Such a model is usually designed with the CAD program and, once the design on the computer is completed, the file is saved in STL format, allowing the 3D printer to obtain the design data. The prototypes of the cellular structures were manufactured in PLA by a 3D printer of the FDM type with a double extruder (nozzle diameter equal to 0.4 mm), which has a printing volume of 230 × 190 × 200 mm^3^. Ten hours is the time required for printing, depending on the set-up of the layer height and the required surface finishing. After that, the PLA model was covered with a mixture of water and plaster specifically employed for metal casting. However, the model cannot be completely encased in plaster without leaving vents as there would be no way for the PLA to be removed and the metal to take its place. Therefore, additional plastic material was added to the model to form the sprues. Once the plastic material that forms the vent channels (the straws) is removed, they will form a tunnel from the outside of the covering to each corner of the model. The model was placed inside a stainless-steel container, which was then filled with plaster and dried. After plaster drying (24 h), the general assembly was placed in the oven for thermal treatment (burning of the PLA model and sprues, 1 h), leaving the final negative shape with a perfect imprint of the design. Meanwhile, in a suitable crucible, the AA6082 was melted using another oven. When PLA burning was completed and the AA6082 became molten, the Al alloy was poured in the stainless-steel container replicating the starting shape of the PLA lattice until solidification of the alloy (5 min). Once at room temperature, the final Al lattice was pulled out from the plaster. A sketch of the manufacturing process is shown in Figure 1a–f. Further details regarding the optimization of the manufacturing process are described in earlier works of the authors [12,13,14].

By using this production process and appropriately setting the parameters of interest on the CAD program, it is possible to reproduce a great variety of geometries knowing a priori the theoretical values of the surface and volume required for the structures after printing. The technique is extremely flexible and versatile for the manufacturing of the required cellular structures. In this work, in particular, it was decided to characterize a cellular structure obtained through the coupling of 33 Kelvin cells (truncated octahedron). The number of cells was decided based on the size of the molds available in the laboratory.

### Kelvin Cell Selection

**Figure 2:**
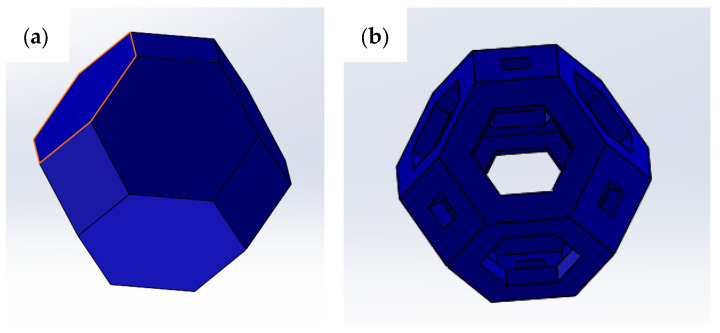
(**a**) Full Kelvin cell; (**b**) hollow Kelvin cell.

**Figure 3:**
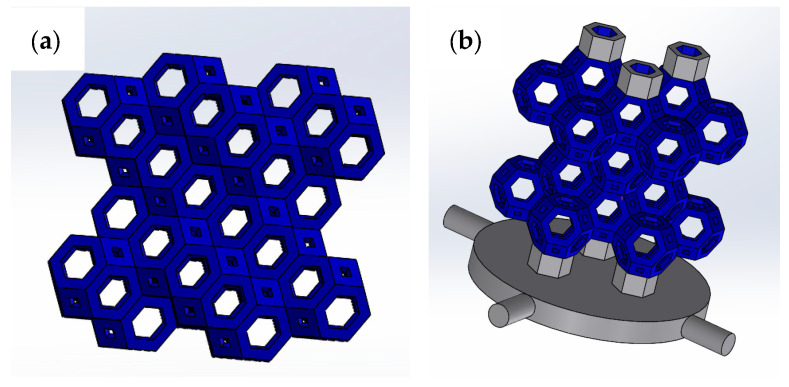
(**a**) Structure of Kelvin cells for compression tests; (**b**) structure of Kelvin cells for compression test with printing support.

Kelvin cells (Figure 2a) are polyhedrons made of fourteen sides, with six quadrilaterals and eight hexagons as faces. For centuries, such a structure has been considered the best way to divide three-dimensional space into cells with the same volume, minimizing the surface area of the cell walls. The Kelvin problem, posed by Lord Kelvin in 1887, is to find an arrangement of cells so that the total surface area of the walls between them is as small as possible. The problem is relevant in nature, for example, for bone replacement materials due to the honeycomb-like structure of the bones. Kelvin suggested the solution to his problem as a structure with six square faces and eight hexagonal faces, named truncated octahedron (Figure 2b). The structure designed for compression tests is reported in Figure 3a, while the structure built-up with the support for printing is reported in Figure 3b.

To create the structure, it was necessary to use both extruders of the 3D printer present in the laboratory as the sample to be reproduced has oblique edges and therefore requires supports, in PVA, to be obtained without the presence of defects.

The following parameters were chosen for samples printing, reported in Table 1:

- Speed 40 mm/s (PLA) and 60 mm/s (PVA);
- Temperature 200 °C (extruders) and 60 °C (printing plate);
- Layer thickness 0.25 mm;
- Fill density 20%.

**Table 1:** Material weight for printing samples.

Sample	PLA [m]	PVA [m]	Weight PLA [g]	Weight PVA [g]	Weight Tot [g]
Kelvin	2.53	4.69	20.0	37.4	57.4

The relative density ρ* for a cellular structure is defined as the ratio between the density of the cellular structure ρ*_cell_* and that of the solid filled structure ρ*_s_*:

(1) $$\rho^{*}=\frac{\rho_{cell}}{\rho_{s}}=\frac{m_{cell}}{m_{s}}$$

Since the densities are calculated for the same unit volume, it is possible to express ρ* also as a ratio of the masses, and since the material does not change, it is possible to express ρ* also as a ratio between the volumes. By exploiting this last relationship, it is possible to easily obtain the value of theoretical ρ* (Table 2). S is the external surface of the lattice structure calculated with the CAD program.

After printing, the PVA support was removed; the sample was left in water at room temperature for approximately 5 h; and then, the PLA model was left to dry. Once the PLA sample was dry, the straws were inserted, and the mixture of water and plaster could be prepared inside the mold. Following the instructions of the plaster manufacturer, a mixture of water and plaster with a ratio of approximately 40:100 was prepared. After the mixture reached the right consistency, it completely covered the sample, leaving only the casting channels and the straw vents free. Once this step was completed, the assembly was left to dry for at least 24 h.

When the plaster mold was dry, the oven was turned on and the mold was inserted into it until 900 °C was reached. The aluminum alloy billet, EN AW 6082, was inserted into another furnace, setting a final temperature of 875 °C. When both ovens reached the pre-established temperatures, it was possible to proceed with casting by applying vibrations to the mold to encourage gas escape and avoid clogging of the casting channels.

As soon as the melt solidified, the sample was quickly immersed in water. Once cooled, the model was cleaned by removing the excess plaster and eliminating the bases and allowances formed during casting. All samples were subjected to compression tests with a tensile–compression MTS Machine (maximum load 50 kN). Each manufactured sample was inserted between compression plates, preloaded at 100 N; after that, compression tests were carried out with the following parameters: crosshead speed 6 mm/min, sampling frequency 10 Hz, and maximum applied load (final densification) 48 kN.

**Figure 4:**
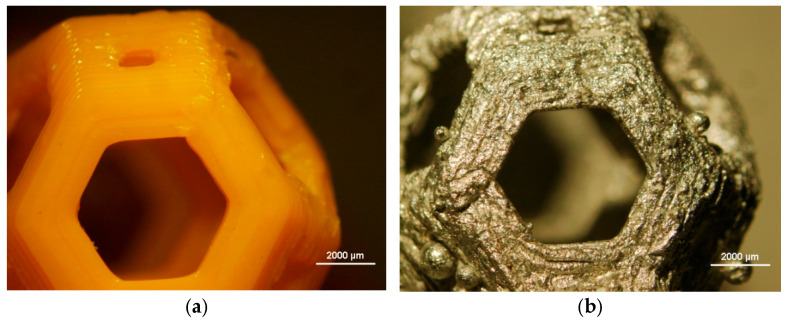
(**a**) Macrograph of Kelvin cells in PLA 3D-printed samples. (**b**) Macrograph of Kelvin cells in AA6082 cast samples.

Both the printed and cast samples are analyzed with a stereo microscope. Pictures are reported on Figure 4. As shown from the images, the casting process faithfully reproduces the details of the surface morphology.

## 3. Results

**Figure 5:**
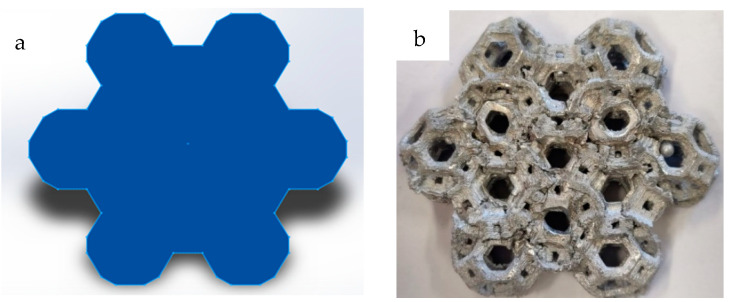
(**a**) Identification of the resistant section for Kelvin cell specimens; (**b**) top view of the manufactured AA6082 samples.

In this work, six samples with the same geometry (Kelvin cell) were manufactured with the lost-PLA technique. The sample weights are reported in Table 3, sizes and geometries are summarized in Table 4. From compression tests, load–displacement data were acquired as raw data. Stress–strain curves were obtained for all samples, dividing the load by the section and the displacement by the starting height of the foams. To calculate the σ-ε curves, it is necessary to identify the resistant section of the samples. Due to the geometry of the Kelvin cell, the specimens were subjected to variations in the resistant section during the compression test. For this reason, an increased area equal to the area subtended by the perimeter of the structure was considered. By using an overestimate of the resistant section (Figure 5), it was possible to safely underestimate the values of σ and the values of absorbed energy. Taking into account the properties of the material, it was possible to calculate the theoretical weight of the structure after casting.

As evidenced from the data in Table 3, it is evident that the printing and casting processes introduce an average error up to 15% in the final weight. The best filling was achieved for sample n. 1, while n. 5 was damaged during the cleaning phase, losing part of the three upper cells. After the compression tests, the samples were reweighed and in five out of six samples, and a minimal weight loss was found due to the detachment of debris from the structure.

**Figure 6:**
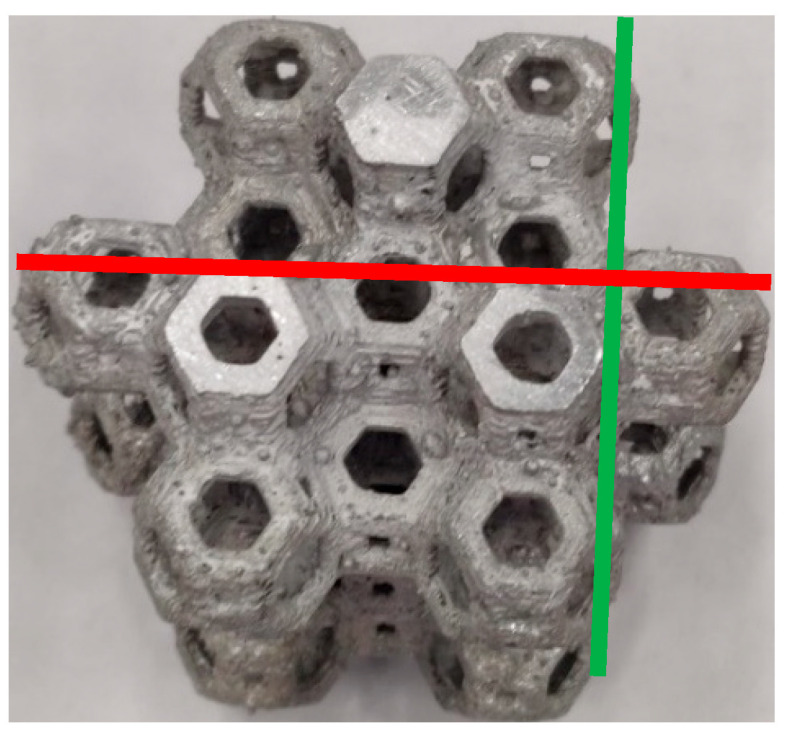
L_x_ (red) and L_y_ (green) identification for the Kelvin cell structures.

The following symbology was used in the subsequent tables (Figure 6):

- L_x_ = maximum length of the specimen along x;
- L_y_ = maximum length of the specimen along y;
- H_i_ = initial height of the specimen;
- H_f_ = final height of the specimen;
- W_i_ = initial weight of the specimen;
- W_f_ = final weight of the specimen;
- SR = resistant section.

The removal of the bases was carried out using a cutting machine; therefore, the difference in the height values of the samples can be attributed to the precision of the cut. All Kelvin cell samples, intact and otherwise, were compressed in the same direction in which the PLA prototypes were printed by the 3D printer; therefore, the planes of the layers were orthogonal to the applied load. The geometrical characteristics and weight before and after the compression tests are reported in Table 4 and Table 5, respectively.

**Figure 7:**
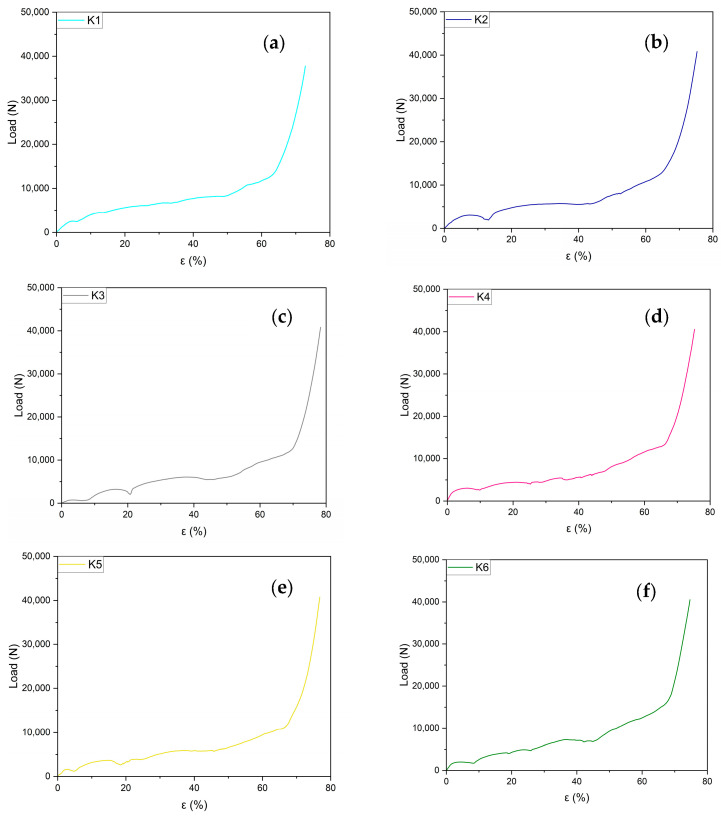
(**a**–**f**) Compression test results for six Kelvin cell structures.

**Figure 8:**
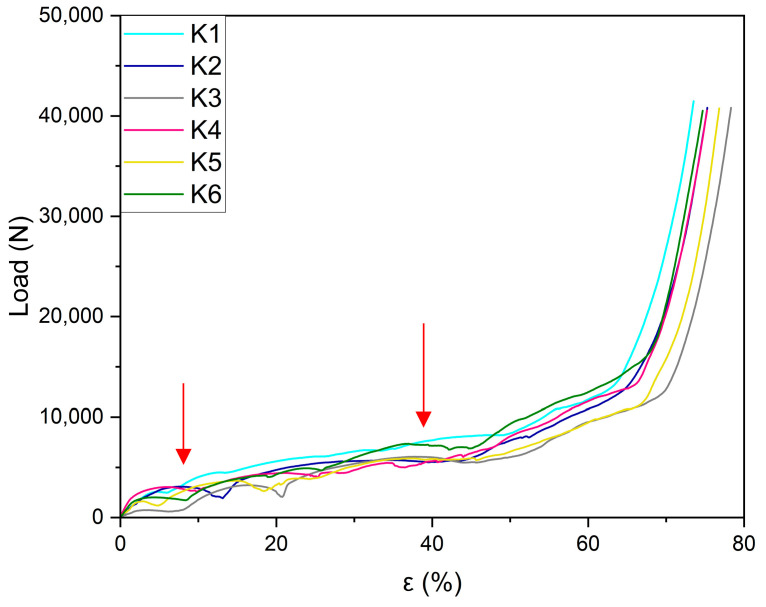
Load–strain curves (comparison) for the six manufactured Kelvin cell structures. The red arrows indicate the areas where the plateaus occurred on average for the six analyzed samples.

**Figure 9:**
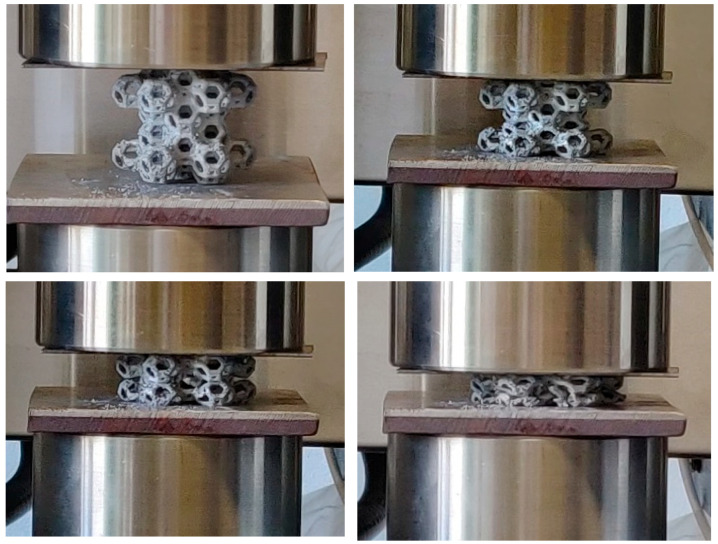
Successive compression steps of the Al Kelvin cell structure.

Compression load–strain graphs for the six Kelvin cell structures are individually reported in Figure 7a–f in order to show, with greater details, the whole trend. All manufactured samples exhibit similar trends for their compression curves. After the initial linear part of the curve, a huge plateau-like part has been evidenced, in which the load is nearly constant or slowly growing. The extension of the plateau stage is up to 65–70% of the total strain. All curves show more or less evident load variation (fall of load) ascribable to the deformation method of the structure and the plastic collapse occurring on the weaker planes, followed by further successive growth of the load. In Figure 8, the whole comparison of all the curves is reported; in Figure 9, photograms at successive compression steps are reported.

## 4. Discussion

As can be observed from Figure 8 good repeatability of the load–strain graphs in the six manufactured samples has been achieved. As a consequence of that, except for some small fluctuations in the load associated with the progressive strain of the structure, the behavior of the material is clearly predictable. After the first linear stage, a huge plateau load at a nearly constant value extends up to 65% of strain; after that, a sudden increase in the load shows that final densification is occurring, with the final strain up to 74%. The results from the tensile tests are reported in Table 6, specifically, the first plateau load (L_p1_) and the second plateau load (L_p2_). In the same table, corresponding loads (L_p1_ and L_p2_) have been reported. These results are in good agreement with the results of the study by Gong et al. [45]. According to this work, Kelvin cell foams under compressive load exhibit cell ligament buckling. As a consequence of that, the mechanical behavior is dependent on anisotropy of the structure and multiaxiality of the applied loads. In all considered samples, a more or less extended post-buckling response has been highlighted, and two plateaus can be easily identified. Their values are reported in Table 6.

The specific energy absorbed before reaching the final densification was then calculated. The value was obtained by considering the area subtended by the σ-ε curve up to 60% for all samples. The results are reported in Table 7 in terms of specific absorbed energy (single value), average value, and standard deviation.

## 5. Conclusions

Kelvin cell AA 6082 cellular structures have been manufactured using the lost-PLA replication method, a simple and economical way for producing metal porous structures. Such a process is extremely flexible and versatile, allowing the parameters of interest to be set in the CAD program and a great variety of geometries to be reproduced. For such manufactured porous structures, mechanical characterization has been performed using static compression tests on six samples with the same geometry. From the analysis of the load–strain curves, the following conclusions can be drawn:

(1) The geometrical characteristics and weight for the Kelvin cellular structures exhibit good repeatability of the manufacturing process for all samples. The theoretical geometric and physical values (from CAD) and the measured ones are in good agreement with each other;
(2) Compression tests carried out in static condition show the classic behavior of an open-cell cellular structure, characterized by a linear stage at the beginning, followed by two plateaus at increasing load, in which the planes collapse while plastic deformation proceeds. Final densification occurs when there is no more space available for further plastic deformation, and a sharp increase in the compressive load is evident;
(3) Load–strain curves show good repeatability and evidence that this manufacturing technique can be easily reproduced and the production process is stable;
(4) Specific absorbed energy values are almost well distributed in the six tests, with a relative standard deviation lower than 15%.

Further developments of this research will involve manufacturing different kinds of open-cell structures (i.e., Weaire–Phelan) with the same lost-PLA technique in order to make a comparison between them in terms of mechanical behavior and energy absorption using compressive tests. Experiments are underway and will be the focus of subsequent work.

## Figures and Tables

**Table 2:** Ideal sizes obtained for the samples.

Sample	V [mm^3^]	S [mm^2^]	S/V [mm^−1^]	ρ* Theoretical	ρ* Theoretical [%]
Kelvin	9139.5	23,920.1	2.62	0.277	27.7

**Table 3:** Weight and relative density of the Kelvin cellular structures.

Samples	Weight [g]	Weight CAD [g]	ΔW [g]	ΔW [%]	$\bar{\Delta W}\;[\%]$	r/r_o_ [%]
K1	22.8	24.6	1.8	7	15	25.7
K2	20.7		3.9	16		23.3
K3	20.7		3.9	16		23.3
K4	21.5		3.1	13		24.2
K5	18.2		6.4	26		20.5
K6	21.2		3.4	14		23.9

**Table 4:** Geometrical characteristics and weight for the Kelvin cellular structures.

Samples	L_x_ [mm]	L_y_ [mm]	H_i_ [mm]	S_R_ [mm^2^]	W_i_ [g]	r/r_o_ [%]
K1	48.4	42.4	40.4	1409.1	22.8	25.7
K2	48.0	42.4	39.3		20.7	23.3
K3	47.8	42.6	42.0		20.7	23.3
K4	48.2	43.1	43.0		21.5	24.2
K5	48.0	41.8	41.2		18.2	20.5
K6	48.1	42.7	41.6		21.2	23.9

**Table 5:** Characteristic sizes for Kelvin samples after the compression tests.

Samples	H_f_ [mm]	ΔH [mm]	ε [%]	$\bar{\varepsilon }$ [%]	W_f_ [g]	ΔW [g]	$\bar{\Delta W}$ [g]
K1	11.4	−29.0	−72	−74	22.6	−0.2	−0.1
K2	10.3	−29.0	−74		20.4	−0.3	
K3	10.2	−31.8	−76		20.6	−0.1	
K4	11.1	−31.9	−74		21.3	−0.2	
K5	10.1	−31.1	−75		18.1	−0.1	
K6	10.8	−30.8	−74		21.2	−0.0	

**Table 6:** Plateau load and strain values in compression tests for KC structures.

Samples	L_p1_ [N]	ε_p1_ (%)	L_p2_ [N]	ε_p2_ (%)
K1	2536	4.5–6.5	8173	43.1–50.3
K2	3100	5.4–10.3	5636	26.5–45.0
K3	3241	12.0–19.4	6059	30.2–41.4
K4	3100	10.2–24.3	5495	33.8–45.5
K5	3664	7.4–16.7	5777	30.2–46.7
K6	2114	1.9–7.3	7327	35.6–45.5

**Table 7:** Specific absorbed energy in the Kelvin cell structure.

	K1	K2	K3	K4	K5	K6
E_spec_ [J/cm^3^]	2.73	2.27	1.92	2.29	2.04	2.55
$\bar{E_{spec}}$ [J/cm^3^]	2.3					
Dev stand	0.3					

## Data Availability

All relevant data are presented within this paper.

## References

[1] Bienvenu Y. (2014). Application and Future of Solid Foams. Comptes Rendus. Phys..

[2] Costanza G., Solaiyappan D., Tata M.E. (2023). Properties, Applications and Recent Developments of Cellular Solid Materials: A Review. Materials.

[3] Song Q., Shi J., Chen X. (2023). Acoustic Emission Characterization Analysis of Quasi-Static and Fatigue Compression Properties of Aluminum Foam. Processes.

[4] Bhuvanesh M., Costanza G., Tata M.E. (2023). Research Progress on Mechanical Behavior of Closed-Cell Al Foams Influenced by Different TiH2 and SiC Additions and Correlation Porosity-Mechanical Properties. Appl. Sci..

[5] Samuel A.M., Samuel E., Songmene V., Samuel F.H. (2023). A Review on Porosity Formation in Aluminum-Based Alloys. Materials.

[6] Hassan A., Alnaser I.A. (2024). A Review of Different Manufacturing Methods of Metallic Foams. ACS Omega.

[7] Costanza G., Gusmano R., Montanari M.E. (2003). Tata Manufacturing Routes and Application of Metals Foams. Metall. Ital..

[8] Parveez B., Jamal N.A., Anuar H., Ahmad Y., Aabid A., Baig M. (2022). Microstructure and Mechanical Properties of Metal Foams Fabricated via Melt Foaming and Powder Metallurgy Technique: A Review. Materials.

[9] Costanza G., Tata M.E. (2011). Metal Foams: Recent Experimental Results and Further Developments. Metall. Ital..

[10] Wu Y., Fang J., Wu C., Li C., Sun G., Li Q. (2023). Additively Manufactured Materials and Structures: A State-of-the-Art Review on Their Mechanical Characteristics and Energy Absorption. Int. J. Mech. Sci..

[11] Kalia K., Ameli A. (2024). Additive Manufacturing of Functionally Graded Foams: Material Extrusion Process Design, Part Design, and Mechanical Testing. Addit. Manuf..

[12] Costanza G., Del Ferraro A., Tata M.E. (2022). Experimental Set-Up of the Production Process and Mechanical Characterization of Metal Foams Manufactured by Lost-PLA Technique with Different Cell Morphology. Metals.

[13] Ceci A., Costanza G., Savi G., Tata M.E. (2024). Optimization of the Lost PLA Production Process for the Manufacturing of Al-Alloy Porous Structures: Recent Developments, Macrostructural and Microstructural Analysis. Int. J. Lightweight Mater. Manuf..

[14] Costanza G., Tata M.E., Trillicoso G. (2021). Al Foams Manufactured by PLA Replication and Sacrifice. Int. J. Lightweight Mater. Manuf..

[15] Orbulov I.N., Szlancsik A., Kemény A., Kincses D. (2022). Low-Cost Light-Weight Composite Metal Foams for Transportation Applications. J. Mater. Eng. Perform.

[16] Sequino L., Capasso C., Costanza G., Tata M.E. (2023). Experimental Investigation on Thermal Effects of a Metal Foam-Based Frame Application for Lithium-Ion Cells. SAE Int. J. Adv. Curr. Pract. Mobil..

[17] Ghiani C., Linul E., Porcu M.C., Marsavina L., Movahedi N., Aymerich F. (2018). Metal Foam-Filled Tubes as Plastic Dissipaters in Earthquake-Resistant Steel Buildings. IOP Conf. Ser. Mater. Sci. Eng..

[18] Kalpakoglou T., Yiatros S. (2022). Metal Foams: A Review for Mechanical Properties under Tensile and Shear Stress. Front. Mater..

[19] Grilec K., Marić G., Jakovljević S. (2010). A Study on Energy Absorption of Aluminium Foam. BHM Berg Hüttenmännische Monatshefte.

[20] Hassanli F., Paydar M.H. (2021). Improvement in Energy Absorption Properties of Aluminum Foams by Designing Pore-Density Distribution. J. Mater. Res. Technol..

[21] Eifert H., Yu C., Banhart J., Baumeister J. (1999). Weight Savings by Aluminum Metal Foams: Production, Properties and Applications in Automotive. Training.

[22] Nawaz A., Rani S. (2021). Fabrication and Evaluation of Percent Porosity and Density Reduction of Aluminium Alloy Foam. Mater. Today Proc..

[23] Lu T., Hess A., Ashby M. (1999). Sound Absorption in Metallic Foams. J. Appl. Phys..

[24] Rajak D.K., Gupta M., Rajak D.K., Gupta M. (2020). Acoustic, Damping, Thermal and Electrical Properties of Metal Foams. An Insight into Metal Based Foams: Processing, Properties and Applications.

[25] Deshmukh S., Kumar S., Wagh A., Krishnan S., Ramamoorthy S. (2022). Selection of Periodic Cellular Structures for Multifunctional Applications Directly Based on Their Unit Cell Geometry. Int. J. Mech. Sci..

[26] Costanza G., Giudice F., Sili A., Tata M.E. (2021). Correlation Modeling between Morphology and Compression Behavior of Closed-Cell Al Foams Based on X-Ray Computed Tomography Observations. Metals.

[27] Brugnolo F., Costanza G., Tata M.E. (2015). Manufacturing and Characterization of AlSi Foams as Core Materials. Procedia Eng..

[28] Al-Ketan O., Rowshan R., Abu Al-Rub R.K. (2018). Topology-Mechanical Property Relationship of 3D Printed Strut, Skeletal, and Sheet Based Periodic Metallic Cellular Materials. Addit. Manuf..

[29] García-Moreno F. (2016). Commercial Applications of Metal Foams: Their Properties and Production. Materials.

[30] Iandiorio C., Mattei G., Marotta E., Costanza G., Tata M.E., Salvini P. (2024). The Beneficial Effect of a TPMS-Based Fillet Shape on the Mechanical Strength of Metal Cubic Lattice Structures. Materials.

[31] Gölbaşı Z., Öztürk B., Beköz Üllen N. (2024). The Structural and Mechanical Properties of Open-Cell Aluminum Foams: Dependency on Porosity, Pore Size, and Ceramic Particle Addition. J. Alloys Compd..

[32] Srivastava S.K., Gupta G.K., Joshi T.C., Rajak D.K., Sanskrita S.K.J., Mondal D., Srivastava A.K. (2023). Insight into Classifications and Manufacturing Processes of Metallic Foams: A Review. Proc. Inst. Mech. Eng. Part E J. Process Mech. Eng..

[33] Bock J., Jacobi A.M. (2013). Geometric Classification of Open-Cell Metal Foams Using X-Ray Micro-Computed Tomography. Mater. Charact..

[34] Liu H., Chen L., Jiang Y., Zhu D., Zhou Y., Wang X. (2023). Multiscale Optimization of Additively Manufactured Graded Non-Stochastic and Stochastic Lattice Structures. Compos. Struct..

[35] Benedetti M., Du Plessis A., Ritchie R.O., Dallago M., Razavi N., Berto F. (2021). Architected Cellular Materials: A Review on Their Mechanical Properties towards Fatigue-Tolerant Design and Fabrication. Mater. Sci. Eng. R Rep..

[36] The Properties of Foams and Lattices. Philosophical Transactions of the Royal Society A: Mathematical, Physical and Engineering Sciences. https://royalsocietypublishing.org/doi/full/10.1098/rsta.2005.1678.

[37] Queheillalt D.T., Wadley H.N.G. (2005). Cellular Metal Lattices with Hollow Trusses. Acta Mater..

[38] Wadley H. (2003). Fabrication and Structural Performance of Periodic Cellular Metal Sandwich Structures. Compos. Sci. Technol..

[39] Lu T., Valdevit L., Evans A. (2005). Active Cooling by Metallic Sandwich Structures with Periodic Cores. Progress Mater. Sci..

[40] Wadley H.N.G., Queheillalt D.T. (2007). Thermal Applications of Cellular Lattice Structures. Mater. Sci. Forum.

[41] Wang N., Kaur I., Singh P., Li L. (2021). Prediction of Effective Thermal Conductivity of Porous Lattice Structures and Validation with Additively Manufactured Metal Foams. Appl. Therm. Eng..

[42] Valdevit L., Pantano A., Stone H.A., Evans A.G. (2006). Optimal Active Cooling Performance of Metallic Sandwich Panels with Prismatic Cores. Int. J. Heat Mass Transf..

[43] Fiedler T., Movahedi N., Stanger R. (2024). On the Efficiency of Air-Cooled Metal Foam Heat Exchangers. Metals.

[44] Konduru R.N., Farges O., Schick V., Hairy P., Gaillard Y., Parent G. (2024). Experimental and Numerical Investigation of Porous Heat Exchangers with Kelvin Cell Structured Foam at High Temperatures: Coupled Conduction-Convection and Radiation Heat Transfer. Int. J. Heat Mass Transf..

[45] Gong L., Kyriakides S., Triantafyllidis N. (2005). On the Stability of Kelvin Cell Foams under Compressive Loads. J. Mech. Phys. Solids.

